# Social and cultural determinants of health; understanding the persisting Alcohol Use Disorder (AUD) in the rural populations in central Kenya

**DOI:** 10.3934/publichealth.2019.4.600

**Published:** 2019-12-23

**Authors:** Danny Mungai, Ronnie Midigo

**Affiliations:** 1 Great Lakes University of Kisumu, Kenya; 2 University of Nairobi, Kenya

**Keywords:** alcohol use disorder, socio-cultural, non-communicable disease

## Abstract

Excessive alcohol use is a significant public health problem globally. Alcohol use typically begins in adolescence or early adult life, and effective prevention strategies focused on this age group are needed to avoid development of Alcohol Use Disorder (AUD). AUD is a worldwide problem resulting in millions of deaths, including hundreds of thousands of young lives lost. It is not only a causal factor in many diseases, but also a precursor to injury and violence. Furthermore, its' negative impacts can spread throughout a community or a country, and beyond, by influencing levels and patterns of alcohol consumption across borders [1]. This study sought to ascertain the influence of socio-cultural factors in AUD among adults. The study adopted a descriptive cross-sectional study design. Stratified random sampling techniques were used to sample alcohol users across the county. Both descriptive (frequencies and percentages) and inferential (chi-square test) statistics were employed in data analysis. Content analysis was used to identify emerging themes in the interviews conducted. The study established that 65% of alcohol users in Muranga County have symptoms of AUD. Socio-cultural factors were found to influence AUD. Based on the findings, it was recommended that the Ministry of health and NACADA should organize sensitizations and awareness drives on alcohol abuse on the worrying trends of AUD together with their associated morbidities. The study also recommended deliberate efforts towards implementation of sound policies aimed at curbing the growth of the AUD.

## 1. Introduction

Alcohol Use Disorders (AUD) affect approximately 76 million people worldwide and about half a million people in Sub Saharan Africa [2] According to Global status report on alcohol and health 2018 [3], the harmful use of alcohol is one of the leading risk factors for population health worldwide and has a direct impact on many health-related targets of the Sustainable Development Goals (SDGs), including those for maternal and child health, infectious diseases (HIV, viral hepatitis, tuberculosis), none communicable diseases and mental health, injuries and poisonings. Alcohol production and consumption is highly relevant to many other goals and targets of the 2030 Agenda for Sustainable Development. Alcohol per capita consumption per year in liters of pure alcohol is one of two indicators for SDG health target 3.5—“Strengthen the prevention and treatment of substance abuse, including narcotic drug abuse and harmful use of alcohol”. Particularly alcohol dependence is associated with a high disease burden and with mortality: about two-thirds of all alcohol-related mortality is caused by the 4% of alcohol users with a diagnosis of alcohol dependence [4]. Therefore, prevention and treatment of, especially severe, AUD should be considered a public health priority. In order to plan prevention and treatment, information is needed about AUD, their course and their risk indicators in the general population. However, current knowledge is strongly skewed because of the emphasis of research on AUD in clinical samples, i.e. the subgroup of people who entered treatment and often have very severe AUD and serious comorbidity. However, most people with an alcohol use disorder do not enter treatment [5]. Although longitudinal population-based research is costly and complex, it is crucial to increase our understanding of demographic and social cultural characteristics of AUD in the general population, such as age, sex, religion, presence of parents of the disorder, level of impairment, consumption level and comorbid psychopathology.

Notably, the few existing community studies suggest that AUD in the general population are generally milder than in clinical samples and that valid notions in clinical samples may not be true in the general population (e.g. an alcohol use disorder is inherently related to excessive drinking; an AUD is a chronic illness; all people with an AUD need treatment) [6]. Hence, besides identification of those groups in the general population that are more likely to develop alcohol problems, examination of the disorder itself in the general population is crucial. Among others, these studies should investigate the following questions: to which degree are AUD related to the level of alcohol intake, what determines whether individuals reach (stable) remission while others do not, and is treatment seeking related to the level of drinking or the severity of the AUD? Therefore, this thesis maps the onset, course and treatment of AUD in the general population. It examines potential risk indicators of a severe or persistent disorder with specific consideration for possible effects of the level of alcohol intake.

### 1.1. Screening tools for AUD

Various screening instruments have been developed to measure alcohol intake and diagnose AUD. The most frequently used screening tool is the Alcohol Use Disorder Identification Test (AUDIT) [6]. The quantity and frequency of alcohol intake is based on self-reports involving calendar methods, particularly the alcohol Timeline Follow Back calendar (TLFB) [7]. The Mini International Neuropsychiatric Interview questionnaire (MINI), based on DSM IV/ICD 10, is a recommended tool for clinical assessment of 13 psychiatric conditions including AUD, however, this tool has to be administered by trained medical personnel; MINI is a gold standard for the diagnosis of AUD in the context of clinical psychiatric assessments [6]. Other tools include AUDIT-C, the Single Alcohol Use Screening Question (SASQ), CAGE4 and FAST5 [8]. Most of these tools have been developed, validated, and are widely used in developed world settings. The Alcohol Use Disorders Identification Test (AUDIT), a self-report alcohol screening tool for excessive drinking developed by WHO, has been used in both high and low income countries and recommended for use in primary care settings among adults [9]. A shorter version of AUDIT, the AUDIT-C that includes the first three questions of AUDIT on alcohol consumption is effective in AUD screening [9].

The Time Line Follow Back (TLFB) calendar method that also relies on self-reported information (in terms of quantity and frequency) has been mainly applied in high-income settings [8]. Because AUDIT and TLFB have been shown to be useful tools for alcohol screening in young people in some settings [9], they are potentially useful to inform alcohol interventions among young people in Africa as well; however, they have not yet been validated among such populations.

### 1.2. Extent of alcohol consumption in Kenya

An addiction to alcohol is known to wreak havoc on the body and negatively affect the life of the individual and the lives of those he or she loves. In Kenya, it appears to have a marked effect, creating dysfunctional and emotionally stunted families. Central region has a history of excessive alcohol consumption and idleness due the high unemployment rate that hits the area [7],[10]. The situation has gone from bad to worse: the women in the province have staged several protest demonstrations in a bid to stop brewers from selling alcoholic drinks to their husbands and sons who have become economically and socially unproductive because of spending most of their valuable time drinking alcohol instead of engaging in other productive activities. A prominent cabinet minister was reported to have suggested that men from other provinces be shipped in to help impregnate the women as the local men could no longer reproduce: replacing one social issue with another.

Alcoholism is not only rampant in Muranga County, but it is a growing concern in the area due to the many cases of marital irresponsibility, social crimes, and other illegal acts that have soared among the alcohol addicts in the area that have raised the concern. For instance, the bar owners continue to report strong revenues as customers are guaranteed drunkenness every night. While consistent drinking in bars appears to cut through ethnicity, region, race and social class, the situation seems worse in Muranga County. While visiting bars is viewed as a social activity in many countries, in Kenya it is purely a male pastime [11]. The purpose is not to socialize or spend time with spouses as done in other countries; it is to drink until the money runs out or the drinker collapses. There is a suggested phenomenon that man who stay home with their families are considered to be “sissies” and insecure: men must visit the bar to asset their masculinity [12]. Whatever the motivation, the reality is that the male obsession with alcohol in Kenya has a far-reaching impact that could be difficult to reverse. There have been numerous cases of young men who lost their lives after consuming tainted alcohol: also called the killer brew. Others have lost their sight as a result of consuming alcohol with methanol. Since many alcohol consumers don't have a steady source of income, they turn to the consumption of lethal illicit brews which have dire physical consequences such loss of sight, healthy problems and in some cases could lead to death.

The main objective of this study was to ascertain the socio-cultural factors determining AUD among the rural population of Muranga County in Kenya. The study was hinged on the tenets of the Social cognitive theory [14] by Bandura. According to the theory, certain behaviors are practiced so long as they could be justified. As such, use of alcohol and the ultimate AUD could be contextualized within culture and justified as such. Going by this argument, users of alcohol develop AUD when they begin to justify their use of alcohol on cultural, environmental and social factors.

## 2. Methodology

This was a descriptive cross-sectional study design utilizing both quantitative and quantitative data collection methods. The study was conducted in Muranga county of Kenya and targeted alcohol users residing within the County. Muranga County is one of the 47 counties in Kenya. According to the Kenya Population and Housing Census of 2009, the county has a population of 942,581. The study focused on all female and male adults aged 18–65 years of sound mind currently using alcohol in Muranga County in Kenya. A total of 385 respondents were sampled based on the Krejcie, Robert, Morgan and Daryle sampling method [12] to participate in the study. AUDIT tool was adopted for the quantitative data while qualitative data was collected using qualitative interview guide based on AUDIT themes.

### 2.1. Demographic characteristic of study population

Demographic factors considered included gender, religion, marital status, employment status, age and availability of parents. Table 1 below presents the demographic characteristics of the respondents.

Of the sampled respondents, about 62.6% were male while 37.4% were female. Christian protestants comprised 67.8% of the sampled population. Those who indicated that they professed Christian catholic religion were 24.2% while those of Islamic religion were 6.2%. About 45.6% of the respondents indicated that they were married, 23.6% single and 16.4% divorced. Majority 42.6% indicated that they had secondary school levels of education. Those with complete primary school education were 11.8% while those with incomplete primary school levels of education were 11.6%. Those with college and university levels of education were 21.2% and 10.4% respectively.

From Table 1, about 32.3% of the sampled respondents indicated that they were employed as casual labourers. Those who were civil servants comprised 25.2% of the respondents while the self-employed were 21.5%. Along age, respondents who were aged between 21–30 years were 25.9%. Respondents aged 31–40 were 33% of the total population while those aged between 41–50 years were 22.9%. Only 6.7% of the respondents were aged 20 years and below. Most of the respondents, 32.3 were casual labourers. Only 21.5% and 25.2% of the respondents indicated that they were self-employed and civil servants respectively. Also 47.4% of the respondents indicated that both their parents were living, 17.9% that their mothers were deceased and 22.3% that their fathers were deceased. Only 12.4% of the respondents indicated that both of their parents were deceased.

**Table 1.** Demographic characteristics of the respondents.

		Frequency	Percent
Gender	Male	239	62.6
Female	142	37.4
Religion	Christian Catholic	92	24.2
Christian protestant	258	67.8
Muslim	24	6.2
No religion	7	1.8
Marital status	Married	174	45.6
Single	90	23.6
Divorced	62	16.4
Widow/Widower	55	14.4
Highest level of education	No formal education	9	2.4
Primary Incomplete	44	11.6
Primary Complete	45	11.8
Secondary	162	42.6
College	81	21.2
University	40	10.4
Employment status	Unemployed	80	21
Civil servant	96	25.2
Self-employed	82	21.5
Casual labor	123	32.3
Age	≤ 20	26	6.7
21–30	99	25.9
31–40	126	33
41–50	87	22.9
51 ≤	44	11.5
Availability of parents	Both parents Living	181	47.4
Mother deceased	68	17.9
Father deceased	85	22.3
Both parents deceased	47	12.4

## 3. Results

### 3.1. Extent of Alcohol Use Disorder

Alcohol Use Disorder was investigated using AUDIT tool. Table 2 below presents the findings. The findings of the AUDIT as indicated in Table 2 above indicates that about 44.6% of the respondents indicated that they took drinks containing alcohol 2–3 times a week. Another 33.9% indicated that their consumption of drinks containing alcohol was 4 or more times a week. On the number of drinks containing alcohol taken on a typical day when drinking, 38.6% and 23.6% of the respondents indicated that they took 3 or 4 and 7, 8 or 9 respectively drinks containing alcohol on typical days. Further, 46.5% of the respondents indicated that they took six or more drinks in one occasion less than monthly while 24.7% indicated that they took six or more drinks on one occasion on a monthly basis.

**Table 2.** Alcohol Use Disorder Identification Test.

		Frequency	Percent
How often do you have a drink containing alcohol?	Monthly or less	18	4.7
2 to 4 times a month	64	16.8
2 to 3 times a week	170	44.6
4 or more times a week	129	33.9
How many drinks containing alcohol do you have on a typical day when you are drinking?	1 or 2	55	14.4
3 or 4	147	38.6
5 or 6	82	21.5
7, 8, or 9	90	23.6
10 or more	8	2.1
How often do you have six or more drinks on one occasion?	Never	53	13.9
Less than monthly	177	46.5
Monthly	94	24.7
Weekly	30	7.9
Daily or almost daily	26	6.8
How often during the last year have you found that you were not able to stop drinking once you had started?	Never	5	1.3
Less than monthly	66	17.3
Monthly	122	32.0
Weekly	75	19.7
Daily or almost daily	113	29.7
How often during the last year have you failed to do what was normally expected from you because of drinking?	Never	119	31.2
Less than monthly	48	12.6
Monthly	86	22.6
Weekly	58	15.2
Daily or almost daily	69	18.1
How often during the last year have you been unable to remember what happened the night before because you had been drinking?	Never	127	33.3
Less than monthly	71	18.6
Monthly	80	21.0
Weekly	89	23.4
Daily or almost daily	13	3.4
How often during the last year have you needed an alcoholic drink first thing in the morning to get yourself going after a night of heavy drinking?	Never	64	16.8
Less than monthly	42	11.0
Monthly	54	14.2
Weekly	149	39.1
Daily or almost daily	72	18.9
How often during the last year have you had a feeling of guilt or remorse after drinking?	Never	8	2.1
Less than monthly	51	13.4
Monthly	77	20.2
Weekly	114	29.9
Daily or almost daily	132	34.6
Have you or someone else been injured as a result of your drinking?	No	138	36.2
	Yes, but not in the last year	85	22.3
	Yes, during the last year	158	41.5
Has a relative, friend, doctor, or another health professional expressed concern about your drinking or suggested you cut down?	No	48	12.6
	Yes, but not in the last year	144	37.8
	Yes, during the last year	189	49.6

Asked to indicate occurrence of situations where they found that they were not able to stop drinking once they started drinking, 32% indicated that this occurred on a monthly basis. About 29.7% indicated that this occurred daily or almost daily. Another 19.7% indicated that this occurred on a weekly basis. About 22.6% and 18.1% respectively of the respondents indicated that they often failed to do what was normally expected from them because on drinking on monthly and daily or almost daily basis. However, 31.2% indicated that this never occurred to them. Further, 23.4% and 21% of the respondents indicated that they failed to remember what happened the night before because you they had been drinking on a weekly and monthly basis respectively. Another 33.3% however indicated that this never occurred.

On occasions when respondents needed alcoholic drinks first thing in the morning to get themselves going after a night of heavy drinking, 39.1% and 18.9% indicated that this occurred on a weekly and daily or almost daily occasions respectively. About 34.6% of the respondents indicated that they had a feeling of guilt or remorse after drinking on a daily or almost daily basis. Another 29.9% and 20.2% of the respondents indicated that this occurred on a weekly and monthly basis respectively.

About 41.5% of the respondents indicated that they or someone else had been injured as a result of their drinking during the last year, 22.3% not in the last year while 36.2% indicated that this never occurred. Further, about 49.6% of the respondents indicated that a relative, friend, doctor, or another health professional expressed concern about their drinking or suggested they cut down during the last year. Another 37.8% indicated that this happened but not in the previous year. Only 12.6% indicated that this never occurred.

**Figure 1.**
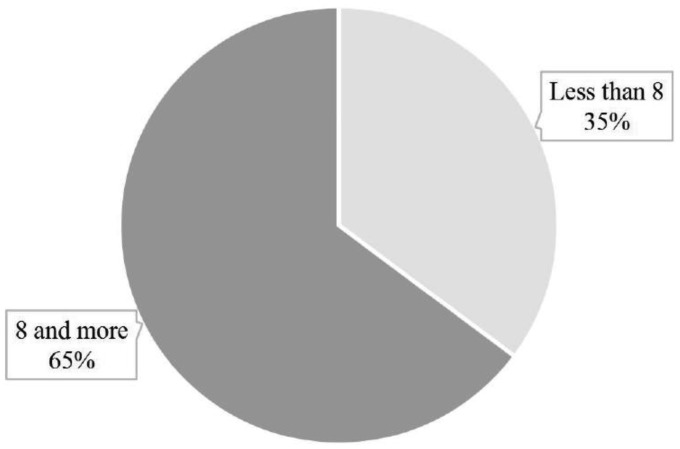
Proportions with AUD.

Following the AUDIT guidelines, scores for individual respondents were computed so as to come up with the percentage of the respondents with AUD. Respondents with 8 or more scores are interpreted as having AUD. Figure 1 below presents the findings

The findings of the study indicate that about 65% of the respondents had scores of 8 or more. Only 35% had scores less than 8.

### 3.2. Socio-cultural factors influencing Alcohol Use Disorder

In order to investigate the socio-cultural factors influencing AUD, respondents were requested to respond to a series of questions indicating their opinion or perceptions. Their responses were cross tabulated against their AUDIT scores. Table 3 below presents the findings

**Table 3.** Socio-cultural factors influencing AUD.

Variable		Scores		Total	CL (95%)	P-value
Less than 8	8 and more
Does your father use alcohol	Yes	53 (28.0%)	136 (72.0%)	189 (49.6%)	1	0.012067
No	81 (40.1%)	121 (59.9%)	202 (53.0%)	0.58 (0.38–0.89)
Does mother use alcohol	Yes	12 (27.3%)	32 (72.7%)	44 (11.5%)	1	0.243387
No	122 (36.2%)	215 (63.8%)	337 (88.5%)	0.66 (0.33–1.33)
Does any of your siblings use alcohol	Yes	34 (37.4%)	57 (62.6%)	91 (23.9%)	1	0.615707
No	100 (34.5%)	190 (65.5%)	290 (76.1%)	1.13 (0.70–1.85)
Any other family members who use alcohol	Yes	32 (35.6%)	58 (64.4%)	90 (23.6%)	1	0.930265
No	102 (35.1%)	189 (64.9%)	291 (76.4%)	1.02 (0.62–1.68)
Was alcohol brewed or available at home	Yes	2 (3.9%)	49 (96.1%)	51 (13.4%)	1	< 0.001
No	132 (40.0%)	198 (60.0%)	330 (86.6%)	0.06 (0.01–0.26)
Is any member of your family struggling with alcohol abuse	Yes	43 (21.1%)	161 (78.9%)	204 (53.5%)	1	< 0.001
No	91 (51.4%)	86 (48.6%)	177 (46.5%)	0.25 (0.16–0.39)
Do cultural beliefs and practices advance usage of alcohol in your community	Yes	22 (9.3%)	214 (90.7%)	236 (61.9%)	1	< 0.001
No	112 (79.4%)	29 (20.6%)	141 (37.0%)	0.03 (0.01–0.05)
Do you think people resort to alcohol use to deal with life stresses	Yes	119 (43.3%)	156 (56.7%)	275 (72.2%)	1	< 0.001
No	15 (14.2%)	91 (85.8%)	106 (27.8%)	4.68 (2.55–8.40)
Does the environment in your community favor the use of alcohol	Yes	64 (30.3%)	147 (69.7%)	211 (55.4%)	1	0.027548
No	70 (41.2%)	100 (58.8%)	170 (44.6%)	0.62 (0.41–0.95)
Peer influence is the cause of alcohol use	Yes	64 (30.0%)	149 (70.0%)	213 (55.9%)	1	0.018358
No	70 (41.7%)	98 (58.3%)	168 (44.1%)	0.6 (0.39–0.92)
Do you think religion restrains alcohol use	Yes	64 (32.8%)	131 (67.2%)	195 (51.2%)	1	0.325297
No	70 (37.6%)	116 (62.4%)	186 (48.8%)	0.81 (0.53–1.23)

The findings of the study as indicated in Table 3 above indicates that use of alcohol by father, brewing of alcohol or availability of alcohol at home and perceptions on positive relationship between cultural beliefs and alcohol abuse were found to be of statistically significant relationship with AUD. Also, the study found that presence of a family members struggling alcohol abuse, opinion that people people resort to alcohol use to deal with life stresses. This was also true of the perceptions that environment and peer influence favor use of alcohol (p < 0.05).

About 72% of the respondents who indicated that their father used alcohol also had AUD. Those whose fathers did not use alcohol were 0.58 times (CL = 0.38–0.89) less likely to develop AUD.

When respondents indicated that alcohol was brewed or available at home, 96.1% of them also hade AUD with an odd of 0.06 for those indicating otherwise. Respondents who indicated that at least a member of their family was struggling with alcohol abuse had 78.9% of them with AUD as compared to 48.6% of them who indicated otherwise. The odds ratio obtained against family members struggling with alcohol was 0.25.

The study also established that when respondents indicated that cultural belief practices do not advance usage of alcohol, they were only 0.03 times more likely to exhibit AUD. On the contrary, about 90.7% of those who indicated that cultural practices advance usage of alcohol had AUD symptoms. Majority (85.8%) of the respondents who indicated a contrary opinion to that that people resort to alcohol use to deal with life stresses had AUD symptoms. The odds of those with the contrary opinion showing symptoms of AUD was established to be 4.68. Further, 69.7% of the respondents who indicated that their environment (community) favored use of alcohol also had AUD symptoms (Odds ratio = 0.62, CL = 0.41–0.95). As to whether peer influence caused alcohol use, 70% of those with similar opinion also had AUD. The odd of contrary opinion was established to be 0.6 (CL = 0.39–0.92).

## 4. Discussion

### 4.1. Extent of Alcohol Use Disorder

The study established that about 65% of alcohol users in Muranga County have symptoms of AUD had scores of 8 or more. Most users of alcohol in the county took drinks containing alcohol 2–3 times a week. They also took 3 or 4 drinks containing alcohol on a typical day when drinking. Such individuals took six or more drinks in one occasion less than. A majority of them on a monthly basis found that they were not able to stop drinking once they started drinking within the previous year. Most of them could remember what happened the night before because you they had been drinking. On a weekly basis, such alcohol users needed alcoholic drinks first thing in the morning to get themselves going after a night of heavy drinking. Also, most of them had a feeling of guilt or remorse after drinking on a daily or almost daily basis. Further, most of the alcohol users in Muranga County indicated that they or someone else had been injured as a result of their drinking during the last year. Finally, most alcohol users in Muranga County had a relative, friend, doctor, or another health professional expressing concern about their drinking or suggesting they cut down during the last year.

These findings lead to an understanding that about 7 out of 10 users of alcohol in Muranga County are suffering from AUD. The Key Informant Interviews also reveal a possibility of high percentages of alcohol users with AUD. In an interview with a NACADA regional officer, it emerged that

*Many people actually suffer from drug and alcohol abuse. Most people in this area have reached a point where they can't function without alcohol. They depend so much on alcohol and the net effect is that they become sick and weak to the extent that they are not able to perform their duties* (KII, NACADA).

The high percentage of individuals with AUD symptoms in the study area is not unique since WHO (2019) had indicated that about 76.3 million are diagnosed with AUD. Growing number of alcohol users could also be a factor contributing to the high number of persons with AUD symptoms.

### 4.2. Socio-cultural factors influencing Alcohol Use Disorder

With regard to the Socio-Cultural factors influencing AUD, this study establishes that Individuals with AUD had the following socio-cultural characteristics:

- Father uses alcohol.
- Alcohol is brewed or available at home.
- Believe that there is a positive relationship between cultural beliefs and alcohol abuse.
- Have family members struggling alcohol abuse.
- Have opinion that people resort to alcohol use to deal with life stresses.
- Perceive environment to be favoring use of alcohol.
- Perceive peer influence to be pushing people to take alcohol.

The Key Informant Interviews conducted also revealed that among other factors, the environment and peer influence influenced AUD. In an interview with a medical officer, it emerged that some people engage in alcohol abuse because it is fashionable to do so and that the environment played a role as well. The medical officer posed that:

*Here in Muranga County, people take alcohol because everyone else is taking it. People meet at the bars to discuss issues affecting them, to run away from stressors and to have time together with friends. In such an environment, it becomes difficult not to drink* (KII, MO).

These findings may lead to an understanding that alcohol users whose fathers are using alcohol are also likely to develop AUD. It is possible to conclude that fathers play a role in regulating uncontrolled behaviors. This finding is in line with the arguments advanced by the psycho-social theory [13] where it is postulated that certain behaviors are either reinforced negatively or positively reinforced by significant people in our lives. Going by this argument, it is possible that alcohol users whose fathers were also using alcohol experienced positive reinforcement in their alcoholic behaviors. The same reasoning could also be advanced for cases where alcohol was brewed or was available at home as well as where a family member was struggling with alcohol abuse.

The study established that beliefs and perceptions justifying taking of alcohol also influenced development of AUD. This finding is hinged on the tenets of the social cognitive theory [14] by Bandura. According to the social cognitive theory, certain behaviors are practiced so long as they could be justified. Going by this argument, users of alcohol develop AUD when they begin to justify their use of alcohol on cultural, environmental and social factors.

## 5. Conclusion and recommendations

The study concludes that about 65% of alcohol users in Muranga County have symptoms of AUD. The socio-cultural factors influencing AUD include fathers of alcohol, brewing or availability of alcohol at home, belief that that there is a positive relationship between cultural beliefs and alcohol abuse, presence of family members struggling alcohol abuse, having opinion that people resort to alcohol use to deal with life stresses, perception of the environment to be favoring use of alcohol and perception of peer influence to be pushing people to take alcohol.

The study revealed that a large proportion of alcohol users in Muranga County have AUD symptoms. The study also established that socio-cultural factors influence AUD. The study recommends other studies to ascertain prevalence of AUD separate for urban and rural areas. Such studies could include other methods for testing alcohol use.

Based on the findings of this study, it is recommended that sensitizations and awareness drives about alcohol abuse could be organized by the Ministry of health and NACADA on the worrying trends of AUD together with their associated morbidities. Such drives could address the demographic and socio-cultural factors associated with AUD. The study also recommends deliberate efforts towards implementation of sound policies aimed at curbing the growth of the AUD in the study population.
